# Variola and Vaccinia

**Published:** 1860-04

**Authors:** Ephraim Cutter

**Affiliations:** Woburn, Mass.


### VARIOLA AND VACCINA.

*Unsuccessful attempts to produce Variola in the Cow, by Inoculating with the Virus of true Variola. Perfect success on using the ordinary Vaccine Virus.* BY EPHRAIM CUTTER, M. D., Woburn, Mass.

Acting upon the commonly received opinion both of the public and the profession—namely, that cow-pox is small-pox modified and mitigated by a transmission through the system of the cow—it would be natural for any physician to expect to procure pure primary vaccine virus by simply introducing into the system of the cow the virus of variola from the human subject.

It was under this conviction that a series of experiments were conducted upon about fifty kine. It is proposed to give an account of the same in the present paper.

*Experiment* 1.—Nov. 26th, 1859. Inoculated four young kine with variola-virus taken on November 19th, from one of my own patients, at about the eighth day of the eruption. Punctures were made with a lancet upon the hairless skin beneath the tail and near the vulva. The virus was introduced upon quills and covered with isinglass plaster, as in the ordinary mode of vaccinating the human subject. At the expiration of a week, no effect like vaccination was produced. In fact, there seemed to be nothing more than a moderate inflammation, just such as would result from a non-specific puncture.

*Experiment* 2.—Dec. 27th. With variolus virus taken on the eighth day (Dec. 22d) of the eruption, by Dr. Luther Parks, Jr., of Boston, Dr. Alonzo Chapin, of Winchester, at my request, inoculated five kine. The writer inoculated seven including steers and heifers.

Punctures were made with a lancet, near the vulva or anus, and upon the teats. The quills, charged with the virus, were introduced, allowed to remain a few minutes, and then suffered to drop out. The operations were conducted with the greatest care, so that there should be no mistake. About five quills were used upon each animal in this as in all the other experiments. On the 31st of December a few of the spots presented to the feel a round and flat hardness, about half an inch in diameter. One spot had a central depression. This promised so much that it was very confidently expected that vaccine virus would be obtained. But the hope was illusory, for the spot did not pass through the normal stages of a vaccine pustule. On the contrary, it remained the same for more than a week, and then faded away. It was suggested by Dr. Chapin that the virus might possibly have been taken from a varioloid patient, (it sometimes being very difficult to distinguish between them,) and that thus the experiments proved nugatory. Subsequent experience, however, has not borne out the supposition.

*Experiment* 3.—Jan. 6th, 1860. Visited a patient of Dr. Toothaker's of Wilmington, sick with severe variola, and charged quills with matter. The eruption was at the seventh day. Inoculated seven kine with this matter, January 6th, 1860. In these cases the cuticle was abraded by scratches, made with a lancet, at right angles to each other, until the serum of the blood began to escape. The charged quill points were then rubbed upon the abrasions for a moment or two

No satisfactory results. To be sure, pustules, or something that looked like pustules, were obtained. They were umbilicated, and some of them hard to the feel, but no lymph could be got.

*Experiment* 4.—Procured some quills charged with variolous virus, from Dr. R. L. Hodgdon, of West Cambridge, on January 6th, and on January 7th inoculated three young kine with the same. The mode was the same as in experiment 3d. No satisfactory result.

*Experiment* 5.—Jan. 13th. Visited, with Dr. Drew, of Woburn, a small-pox patient under his care, and charged some quills in the usual way. Besides I charged some cotton threads, by rupturing pustules and imbuing in the lymph the threads for the distance of half an inch or more at their middle part. Within an hour of the procuring of this virus, the quills were inserted into several cattle by the usual punctures with the lancet. The threads were introduced beneath the skin by means of a needle. They were then drawn through to the point charged with the virus, and with this engaged under the cutis, the ends were tied, and the seton thus formed left in. The threads remained in for three days. Inflammatory action ensued. There was swelling, with soreness in the vicinity of the punctures. Upon removing the threads, however, these symptoms subsided. No normal pustule was produced. This essay was deemed almost an "experimentum crucis."

*Experiment* 6.—Jan. 18th. Went to Lexington and took quills and threads from a small-pox patient of Dr. Currier, in the same manner as was practised in Experiment 5th. These were used in inoculating four cattle on the 20th of January. These essays were without success, although the threads were allowed to remain a week.

*Experiment* 7.—Jan. 25th. Received by express, from Dr. John A. Lamson, of Boston, some variolous virus from one of the crew of the slave yacht Wanderer. This matter was selected with care, and the case was a well developed one. These quills were used upon four or five cattle.

At the expiration of a week, there was one pustule developed out of the sixteen or twenty punctures. Took what seemed to be lymph from this one pustule, and tested it, without success, upon another cow.

It is natural to expect, that after so many careful experiments, conducted without succes, the experimenter should begin to doubt. I questioned my ability to inoculate; but of this I was not convinced, as I had successfully vaccinated a considerable number of human subjects during my practice of medicine. Besides, I was told by some, who had tried the same experiments without success, that it could not be done; that cow-pox must be found in a natural condition.

About this time, Dr. Currier, of Lexington, in conversation with my father, Dr. B. Cutter, stated that within the past ten years he had seen some cases of the cow-pox, occurring in the natural way upon the cow; and he further expressed his belief that such cases must exist in our vicinity at the present time. We should look for them in young cows, just after their second calving, and for the locality of the eruption upon the udder, between the hind teats. One case he mentioned as having about one hundred pock upon a remarkably clear udder. He took a large number of quills from them, and vaccinated three persons with them, viz: himself and two "Irish" infants. He did not succeed in his own case, but the children "took" severely. The remainder of the quills were mislaid, and he lost the opportunity of any further trials.

I was led to examine quite a number of cattle, in different localities, to find this disease in the natural state. I was incidentally surprised to find that a majority of the cattle examined had upon the teats and udder a considerable variety of pustular and horny skin diseases, so that if it is thought (and such is the opinion of the public, I believe) that *purer virus* can be got from the cow than from the human subject, it is an easy matter to explode this idle theory. While conducting Experiment 7th, I found one cow with the hinder part of the udder covered with non-umbilicated pustules of the size and feel of a normal vaccine pustule.

*Experiment* 8.—I took the virus from the most developed of these pustules, and tried it on other cows. No infection followed, thus proving (if the operation was properly performed) that this was not the true vaccine pustule.

*Experiment* 9.—A repetition of Experiment 8th, the matter being taken from a similar pustule near the vulva of another cow, and inserted into another animal. No infection resulted.

*Experiment* 10.—Still another cow was found with a disease upon the udder, simulating the natural disease. This ran its course in about a fortnight. There were pustules, non-umbilicated, full of white lymph. Vaccination with this lymph upon quills was tried upon another cow, and about a week later some of the crusts were rubbed up with water to the consistency of pus, and then pricked into crucial abrasions of the cuticle of a two-days'-old calf, by Dr. Chapin, and of a cow, by myself. These vaccinations have not yet had time to develop themselves. If they do not take, should we not be justified in calling these cases the spurious cow-pox referred to by Jenner?

*Experiment* 11.—Jan. 20th, 1860. Vaccinated four kine with ordinary vaccine, such as I was using in vaccinating the human subject.

Jan. 24th.—The spots all look as if taking.

Jan. 26th.—On two of the four kine, umbilicated pustules, having, in one instance, a whitish summit, and in other instances being more swollen, with summits less white.

Jan. 27th.—One of the kine has three spots, half an inch in diameter. Bluish color, well marked. Took a large number of quills, and on the same evening sent specimens to the members of the Middlesex East Society, and to other physicians who had assisted in procuring variolous matter for the purpose of experiment.

Jan. 28th.—Dr. Chapin visited the animals. He was assured of the abnormal characteristics of the *quasi* pustules procured by inoculation, and was satisfied with the normal appearance of the pustules produced by the vaccination.

*Experiment* 12.—Jan. 23d, 1860. Vaccinated two cows with vaccine virus from a child, on the seton plan. Did not take. Failure probably due to the imperfect moistening of the threads.

*Experiment* 13.—Jan. 24th, 1860. Vaccinated, on the seton plan, four kine, with virus received by mail from Dr. J. D. Mansfield, of South Reading. No other results ensued than what would ordinarily be expected to follow the introduction of an uncharged thread.

*Experiment* 14.—Jan. 28th. Dr. Chapin vaccinated two kine with the virus he ordinarily uses upon the human subject. Both took well, and a large number of quills were obtained from them, which were used with general success.

Since the last experiments, I have often successfully vaccinated kine, both with the crust and the quill. The pustules have generally been large, and have matured upon the eighth or ninth day after vaccination. They vary in size somewhat, being generally very large, and not small. In some, a characteristic blue color of the pustule and vicinity is observed. This happens especially when the seat of vaccination is upon the part of the labium where the skin merges into the mucous membrane. No constitutional effects upon the cows have been noticed.

From the account given in a late number of the *Boston Medical and Surgical Journal*, of the experiments of Dr. Martin, of Attleborough, Mass., some years ago, and the most unfortunate results that followed, I tremble at the risks I have been running, for if quills had been obtained they would have been used. It is only an instance of an overruling Providence. However, I am more confirmed, by Dr. Martin's account, in my opinion, derived from the above-described experiments, that *vaccinia is not varioloid*, but that it is a *distinct affection*. I cannot explain the experiments of Dr. Adams, of Waltham, Mass., nor of Mr. Ceely, nor of the Russian physician, who profess to have succeeded in inoculating cows, and thus procuring the vaccine disease. I only know that Dr. Chapin and myself did not succeed. If some more successful operator should report and annul my assertions, I shall be very happy, as all that I desire is the truth.

Three modes of introducing the variolous matter into kine were used—(inoculation.)

1. By quill and puncture with lancets.

2. By rubbing the charged points of quills upon crucial abrasions of the hairless cutis.

3. By introducing, in the form of setons, threads charged with the variolous virus. This is the easiest and most expeditious way of inoculating or vaccinating kine. There is but one struggle with the animal, and, once in, it stays in.

*Vaccination* on the cow has been practised in the following ways:

1. By the seton; this was tried twice and was not successful.

2. By quills. These, if fresh, generally succeeded.

3. By pricking into crucial abrasions of the cuticle, with a lancet, portions of a scab dissolved in water, until it is of the consistence of a thick paste. This has been uniformly successful upon man or beast; more so than any other mode I have practised.

I think I am justified in asserting that *any one can procure a vaccine pustule on the cow by vaccination*, as easily as it can be procured upon the human subject. This is the mode in which I obtain "*vaccine virus* from the cow."

The question has been asked whether the virus from the cow, thus obtained, is any better than the ordinary virus in use. To this I would reply, than in my opinion it is no better. A few statistics may throw light upon this. Out of nine primary vaccinations noted, with virus from the cow, six took the first time and three the second. Out of fifty-three secondary vaccinations noted, thirty-nine took the first time, thirteen the second time, and one the third time. One of the primary vaccinations taking on the second trial, occurred in a child half an hour old. One of the secondary vaccinations taking on the first trial, occurred in a lady 91 years old, who had been repeatedly vaccinated uncuccessfully. Another of these was vaccinated during the prodomic symptoms of varioloid, and both vaccinia and varioloid ran through their stages contemporaneously.

On the other hand, the writer vaccinated a child with quills from another infant, and they did not take. On the second trial, quills charged with virus from the cow were used. No effect. On the third trial, the third mode of vaccinating, with a scab from the cow, was employed. No result. On the fourth and final trial, the same mode of vaccinating, with a scab from a *child*, succeeded perfectly.

Some of the secondary vaccinations with virus from the cow procured pustules normal in appearance, and of a large size, despite a good scar of the primary vaccination. Constitutional effects have been produced in some cases. However, equally good pustules and similar constitutional effects have been produced, at the same time, by the ordinary virus in use.

Another thing—the ease with which the vaccine pustule is produced in the cow ought to give the profession confidence *in the matter in general use*. Thus the idea that it has "run out" by successive transmissions through human subjects is not supported by the present experiments.

Again, the idea that the vaccine disease is peculiar only to certain districts in Wales is not founded upon fact, as it has been repeatedly observed in this country. It seems idle, then, to send to Europe for vaccine virus, when we have it at our very doors.

To conclude: The object of the present paper has been to show that vaccinia is not varioloid, or cow-pox modified small-pox. It has been attempted to prove this by the unsuccessful attempts to produce a normal vaccine pustule by inoculation, *while upon the very same animals, by vaccination with the virus in ordinary use*, the normal vaccine vesicle has been got easily. According to the authorities consulted, it is still a mooted question in regard to the subject in hand. But Von Bibra says distinctly that the cow-pox and the small-pox are two different diseases. The assserted fact that persons who have had the small-pox have been successfully vaccinated, seems also to substantiate our position.—*Boston Medical and Surgical Journal.*

